# Haplotypes of the endothelial protein C receptor (EPCR) gene are not associated with severe malaria in Tanzania

**DOI:** 10.1186/s12936-015-1007-6

**Published:** 2015-12-01

**Authors:** Helle Holm Hansson, Louise Turner, Line Møller, Christian William Wang, Daniel T. R. Minja, Samwel Gesase, Bruno Mmbando, Ib Christian Bygbjerg, Thor G. Theander, John P. A. Lusingu, Michael Alifrangis, Thomas Lavstsen

**Affiliations:** Centre for Medical Parasitology, Department of Immunology and Microbiology, Faculty of Health and Medical Science, University of Copenhagen, Østerfarimagsgade 5, Building 22-23, 1356 Copenhagen K, Denmark; Department of Clinical Microbiology and Department of Infectious Diseases, Copenhagen University Hospital (Rigshospitalet), Blegdamsvej 9, 2100 Copenhagen Ø, Denmark; Tanga Research Centre, National Institute for Medical Research, Bombo Area, PO Box 5004, Tanga, United Republic of Tanzania

**Keywords:** PROCR, Haplotypes, sEPCR, Severe malaria, Tanzania, Single nucleotide polymorphisms

## Abstract

**Background:**

Endothelial protein C receptor (EPCR) was recently identified as a key receptor for *Plasmodium falciparum* erythrocyte membrane protein 1 mediating sequestration of *P. falciparum*-infected erythrocytes in patients suffering from severe malaria. Soluble EPCR (sEPCR) inhibits binding of *P. falciparum* to EPCR in vitro and increased levels of sEPCR have been associated with the H3 haplotype of the EPCR encoding *PROCR* gene. It has been hypothesized that elevated sEPCR levels, possibly linked to the *PROCR* H3 genetic variant, may confer protection against severe forms of malaria. This study determined the frequencies of *PROCR* haplotypes H1–4 and plasma levels of sEPCR in a Tanzanian study population to investigate a possible association with severe malaria.

**Methods:**

Study participants were children under 5 years of age admitted at the Korogwe District Hospital (N = 143), and diagnosed as having severe malaria (N = 52; including cerebral malaria N = 17), uncomplicated malaria (N = 24), or an infection other than malaria (N = 67). In addition, blood samples from 71 children living in nearby villages were included. The SNPs defining the haplotypes of *PROCR* gene were determined by post-PCR ligation detection reaction-fluorescent microsphere assay.

**Results:**

Individuals carrying at least one H3 allele had significantly higher levels of sEPCR than individuals with no H3 alleles (P < 0.001). No difference in the frequency of H3 was found between the non-malaria patients, malaria patients or the village population (P > 0.1). Plasma levels of sEPCR differed between these three groups, with higher sEPCR levels in the village population compared to the hospitalized patients (P < 0.001) and higher levels in malaria patients compared to non-malaria patients (P = 0.001). However, no differences were found in the distribution of H3 (P = 0.2) or levels of sEPCR (P = 0.8) between patients diagnosed with severe and uncomplicated malaria.

**Conclusion:**

Frequencies of SNPs determining *PROCR* haplotypes were in concordance with other African studies. The *PROCR* H3 allele was associated with higher levels of sEPCR, confirming earlier findings, however, in this Tanzanian population; neither *PROCR* haplotype nor level of sEPCR was associated with severe malaria, however, larger studies are needed to confirm these findings.

## Background

In 2012, there was an estimated 165 million cases of malaria in Africa, leading to over half a million deaths [1]. Severe and fatal cases of malaria are caused by *Plasmodium falciparum* infection [2], and this is precipitated by the ability of this parasite to attach infected erythrocytes to the microvasculature. This sequestration of parasites causes blood occultation and inflammation in host organs, including the brain. The mechanisms resulting in the development of severe malaria have not yet been fully understood, however; recently, the endothelial protein C receptor (EPCR) has been identified as a specific receptor for the *P. falciparum* erythrocyte membrane protein 1 (PfEMP1) variants mediating sequestration of infected erythrocytes in the most severe forms of malaria, including severe anaemia and cerebral malaria [3, 4].

EPCR, also known as activated protein C (APC) receptor plays a key role in the regulation of coagulation, inflammation and endothelial cell integrity [5–9]. It is a transmembrane glycoprotein, mainly expressed on endothelial cells, but also on a variety of other cells, including neutrophils [10–14]. On endothelial cells, EPCR facilitates conversion of circulating plasma zymogen protein C to APC. APC inactivates clotting factors and generates anti-inflammatory and broad cytoprotective effects through protease-activated receptor 1 (PAR-1) signalling [6, 9, 15]. The PfEMP1 and APC binding sites on EPCR overlap and PfEMP1 exhibits a much stronger binding to EPCR than APC. Therefore it is likely that EPCR-mediated parasite sequestration aggravates malaria pathogenesis by disrupting the anti-coagulative, anti-inflammatory and cytoprotective effects of APC [3, 4].

EPCR is also found as a soluble form in the plasma (sEPCR) where it binds APC in competition with cellular bound EPCR [16, 17]. Elevated levels of sEPCR have been associated with single nucleotide polymorphism (SNPs) variations in the human *PROCR* gene, encoding EPCR. *PROCR* variants have been studied mostly in association with venous thrombosis [18–21]. Four haplotypes are defined by the SNPs: H1–H4 [18–20]. The H3 haplotype has been associated with increased plasma sEPCR levels [19, 22, 23] and has been suggested to be a risk factor for venous thrombosis [19, 21], although other studies could not confirm this [18, 20]. Contrary to H3, the H1 haplotype has been associated with elevated APC plasma concentration and a protective effect against venous thrombosis [20, 24]. The H4 haplotype has been associated with a slightly increased risk of venous thrombosis, whereas H2 haplotype has not been associated with increased or decreased risks [18].

Studies investigating the association between *PROCR* haplotype, sEPCR plasma levels and susceptibility to malaria [25–28] have somewhat conflicting conclusions. The A(6936)G SNP-defining H3 was associated with protection from severe malaria in a Thai population [27], whereas no association could be detected between any SNPs in the *PROCR* gene and susceptibility to severe malaria in a Ghanaian study [26]. In a study performed on malaria patients from Benin, higher sEPCR plasma levels were found in patients with cerebral malaria compared to patients with other forms of severe malaria or with uncomplicated malaria, whereas cerebral malaria was associated with lower sEPCR plasma levels in Malawian children [28]. This study set out to determine the frequencies of four EPCR haplotypes and sEPCR levels in an East African Tanzanian study population to elucidate possible associations with severe malaria.

## Methods

### Study population

Study participants were enrolled at the Korogwe District Hospital and from two villages, Kwamasimba and Mkokola, in northeastern Tanzania. From Korogwe District Hospital, children under the age of 5 years were enrolled, if presenting with any symptoms of malaria, after obtaining informed consent from a parent or legal guardian. For diagnostics and research, blood samples were collected and treatment was initiated if the patients were positive by a malaria rapid diagnostic test (mRDT), following national guidelines. Children were considered as having severe malaria if haemoglobin levels were <5 g/dl (severe anaemia), assessed with a Blantyre coma score below 3 (cerebral malaria), showing clinical signs of respiratory distress, or reaching a parasitaemia level above 200.000 asexual stages/µl (hyperparasitaemia). Children not fulfilling the above criteria were defined as having uncomplicated malaria, as previously described [29]. Furthermore, convalescent blood samples from a sub-set of malaria patients were collected after recovery. These samples were randomly selected from a database of the original study of which all the hospitalized patients of this study were part [30, 31].

As part of an ongoing, annual, malariometric, cross-sectional study in the study communities, blood samples were collected from volunteers residing in the villages of Mkokola and Kwamasimba, Korogwe district, northeastern Tanzania in 2007 who tested negative for *P. falciparum* by blood smear microscopy. The study area has previously been described by others [32, 33].

### Determination of the EPCR haplotypes H1, H2, H3, and H4 of the *PROCR* gene

After collection of whole blood, the plasma and buffy coat were each separated from the erythrocytes and stored at −80 and −20 °C, respectively. DNA was extracted from the buffy coat with the NucleoSpin Blood Kit (Machery–Nagel) according to manufacturer’s instructions.

EPCR is encoded by the *PROCR* gene on chromosome 20 and consists of four exons and three introns [34, 35]. Primers for the PCRs were designed to amplify two regions of the *PROCR* gene which define the H1, H3 and H4 haplotypes: the T(4414)C and C(4868)T SNPs in intron 1, and the A(6936)GSNP in exon 4. H1, H3 and H4 were defined based on earlier publications [18, 19], whereas H2 was defined as being none of the above (Table 1) [18, 19]. Size and specificity were tested by agarose gel electrophoreses. For each PCR reaction, 1 µl DNA was amplified in a 10 µl reaction consisting of 1.5 µl H_2_O, 2.5 µl 3 µM primermix, and 5.0 µl TEMPase Hot Start Master (Ampliqon). The reaction was performed in a 96-well PCR plate (Starlab GmbH, Hamburg, Germany) in a VWRi Duo Cycler (VWR/Bie & Berntsen, Radnor, PA, USA).

**Table 1:** Haplotypes of the *PROCR* gene encoding endothelial protein C receptor (EPCR)

EPCR Haplotypes	T(4414)C NA	C(4868)T RS2069948	A(6936)G RS867186
H1	T	*C*	A
H2	T	T	A
H3	T	T	*G*
H4	*C*	T	A

The four haplotypes are determined by three SNPs. Nucleotides defining the haplotype are marked in italics

*NA* not available

Following the PCR reactions, the products were mixed, and 2 µl used in the succeeding analysis; a ligase detection reaction-fluorescent microsphere assay (LDR-FMA). The LDR-FMA was developed similar to a previously described method [36] to analyse the samples for the EPCR haplotypes with minor modifications; 2 µl PCR product was used in the following ligase detection reaction (LDR), added to a 13 µl mastermix consisting of 11.3 µl H_2_O, 0.15 µl 2 µM LDR primermix, 1.5 µl ligase reaction buffer, and 0.05 µl (2 units) Taq DNA ligase (both from New England Biolabs, Beverly, MA, USA). The reaction was performed in a 96-well PCR plate (Starlab GmbH, Hamburg, Germany) in a VWRi Duo Cycler (VWR/Bie & Berntsen). The conditions of the following microsphere assay were modified on a few parameters; 12 µl TMAC (3 M tetramethyl-ammonium chloride, 50 mM Tris–HCl (pH 8.0), 3 mM EDTA (pH 8.0), 0.1 % SDS)/1.2 µl Streptavidin-R-phycoerythrin conjugate (Sigma Aldrich, St Louis, MO, USA) (10 µg/ml) solution was added to each well in the second hybridization; 75 of each microsphere were analysed. All primers were designed based on the GenBank accession number AF106202 and are shown in Table 2 along with reaction conditions and tags complementary to Luminex MagPlex-TAG™ microspheres (Luminex Corp, Austin, TX, USA). Control samples were kindly provided by Dr Shirley Uitte de Willige from the Department of Haematology, Erasmus University Medical Centre Rotterdam, The Netherlands.

**Table 2:** Primers and conditions for the *PROCR* polymerase chain reaction (PCR) and ligase detection reactions (LDR)

	Sequences	Conditions	Microsphere number
*PCR primers*			
Set 1 T(4414)C C(4868)T 647 bp	Fw 5′-CCAGGTTATAATAAGCACTGAATCG-3′ Rv 5′-CTGGTACCACACGTGATAGGG-3′	**95** **°C 15** **min** **30 cycles:** ***95*** ***°C 30*** s ***60*** ***°C 30*** s ***72*** ***°C 60*** s **72** **°C 10** **min**	
Set 2 A(6936)G	Fw 5′-GGCCTATTCTTCGGGCTAAC-3′		
251 bp	Rv 5′-TGGTGTTTCAGTTGGGGAGT-3′		
*LDR primers*			
4414 common	5′-PHOS-TGAGATCATGTCCTTTTCCTACTT-BIO-3′	**95** **°C 1** **min** **40 cycles:** ***95*** ***°C 15*** s ***56*** ***°C 2*** ***min***	
4414 T(A)	5′-ctttctcatactttcaactaatttCTCCACCTGACCCAGGG**A**-3′		20
4414 C (G)	5′-ctttcttaatacattacaacatacCTCCACCTGACCCAGGG**G**-3′		25
4868 common	5′-PHOS-CTTCAGCCTGGGCCG-BIO-3′		
4868 C (G)	5′-catcttcatatcaattctcttattCTGCGGGCAGAGTCA**G**-3′		35
4868 T (A)	5′-tactacttctataactcacttaaaCTGCGGGCAGAGTCA**A**-3′		29
6936 common	5′-PHOS-GTTTCATCATTGCTGGTGTG-BIO-3′		
6936 A	5′-tacttaaacatacaaacttactcaCGTCCTGGTGGGC**A**-3′		65
6936 G	5′-atctcaattacaataacacacaaaCGTCCTGGTGGGC**G**-3′		67

Primer sequences and conditions for the PCR and LDR reactions. For the LDR of T(4414)C and C(4868)T the primers were based on the reverse sequence. Each LDR consists of three primers per SNP: two allele specific primers with anti-TAGs (small letters) matching the TAG sequence on the microsphere (Luminex xTAG microsphere number shown here) and one common primer marked with phosphate (PHOS) at the 5′ end and biotin (BIO) at the 3′ end. The sites of the SNPs are in bold and underscored

### Determination of sEPCR in patient plasma samples

The levels of sEPCR in the patient plasma samples were determined by enzyme-linked immunosorbent assay (ELISA) (Asserachrom, Stago, France) according to manufacturer’s instructions.

### Statistical analysis

Differences in mean age and distribution of gender between the groups were analysed with Mann–Whitney and Chi square test, respectively. The genotype distributions for each SNP were analysed for deviations from the Hardy–Weinberg Equilibrium with The Court Lab Calculator [37]. Chi square test was used to analyse differences in allele or haplotype frequencies. Differences in sEPCR levels were analysed by Mann–Whitney tests. All analyses were done using SigmaPlot 12.3 (SPSS Inc.). P values < 0.05 were considered statistically significant.

### Ethical clearance

Ethical clearance for the study was granted by the Tanzania Medical Research Coordinating Committee and Ministry of Health and Social Welfare, Tanzania. Informed consent was obtained from parents or legal guardians.

## Results

### Clinical and demographical characteristics of study participants

A retrospective selection of samples was collected from children under the age of 5 years, admitted at the Korogwe District Hospital. Seventy-six children were diagnosed with malaria based on mRDTs and positive blood smears, 52 with severe malaria, defined as hyperparasitaemia (N = 12), severe anaemia (N = 19), cerebral malaria (N = 17) or respiratory distress (N = 4). Sixty-seven children were admitted with infections other than malaria (non-malaria group). Blood samples from 71 children without malaria based on mRDTs from two villages in the district, Kwamasimba and Mkokola, were also collected. The demographic characteristics of the study population are presented in Table 3.

**Table 3:** Demographic characteristics of the study participants

	Malaria patients N = 76	Non-malaria patients N = 67	Villages N = 71
Age (years)			
Mean ± SD	2.5 ± 1.2	1.7 ± 1.2	3.8 ± 2.7
Min/max	0.2/5.2	0.1/5.0	1.0/16.2
Gender (n)			
Male	54.7 % (41)	71.2 % (47)	53.1 % (43)
Female	45.3 % (35)	28.8 % (20)	46.3 % (38)

### Determination of H1–4 *PROCR* haplotypes

The specificity of the LDR-FMA methodology was confirmed with control DNA samples from individuals with known genotypes of the SNPs used to determine the four haplotypes. The three SNPs were correctly identified in the control samples, with positive read-outs easily distinguished from the background read-outs in negative control samples. Results were furthermore confirmed by sequencing of DNA from selected patients (N = 24).

The alleles of three SNPs: T(4414)C, T(4868)C and A(6936)G), were determined for 213 individuals and the overall distributions for the alleles were found to be in Hardy–Weinberg equilibrium (4414: Χ^2^ = 0.34, P = 0.6; 4868: Χ^2^ = 0.25, P = 0.6; 6936: Χ^2^ = 0.32, P = 0.6). From the SNP alleles, the four haplotypes were constructed for each individual (Table 1).

### Analysis of the sEPCR levels and the relation to *PROCR* haplotypes

**Fig. 1:**
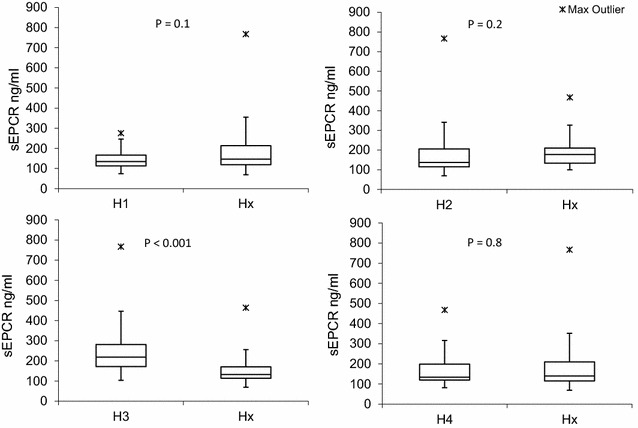
Soluble endothelial protein C receptor (sEPCR) levels of the total study population in the four haplotypes. Hx are defined as all haplotypes but the one given for each of the four analyses. A statistically significant difference was found with higher levels of sEPCR in carriers of at least one H3 allele compared to carriers of none. The significance level was set to P < 0.05

Plasma sEPCR level were measured for each individual and analysed for association to the *PROCR* haplotypes (Fig. 1). Plasma levels of sEPCR were significantly higher in individuals carrying at least one H3 allele [median = 219.1 ng/ml (25/75 quartiles = 171.7/281.6)] compared to individuals with no H3 allele [median 132.9 ng/ml (25/75 quartiles = 113.3/170.5)], P < 0.001. No differences in plasma sEPCR were found between individuals carrying any of the other three haplotypes (P = 0.1, 0.2 and 0.8, for H1, H2 and H4, respectively).

### Analysis of sEPCR level and association with disease

Analysis of H3 distribution in malaria patients, non-malaria patients and village population (Table 4), showed that there was no statistical significant difference between frequency of the H3 allele in the three groups (malaria vs. non-malaria patients: Χ^2^ = 0.03, P = 0.8; malaria patients vs. villages: Χ^2^ = 2.5, P = 0.1; and non-malaria patients vs. villages: Χ^2^ = 2.3, P = 0.1). To determine if high sEPCR plasma levels was associated with malaria, sEPCR levels were compared between the three study groups (Table 5). Malaria patients had higher sEPCR levels [median 161.1 ng/ml, (25/75 quartiles = 124.4/213.4)] than patients with other infectious diseases [median 130.6 ng/ml, (25/75 quartiles = 109.5/165.5)], P < 0.001. The sEPCR levels in the village population were significantly higher [238.0 ng/ml, (25/75 quartiles = 178.0/292.4)] than in the two groups of hospitalized patients (P < 0.001 for both comparisons). In all groups, a significantly higher level of sEPCR was seen in individuals with at least one H3 allele than in those without (P = 0.009, <0.001 and 0.007, for malaria patients, non-malaria patients and village population, respectively) (Table 5).

**Table 4:** Distribution of the four haplotypes in hospitalized patients and village population

Haplotype alleles (N)	Malaria patients N = 76	Non-malaria patients N = 67	Villages N = 71
H1	15.8 % (24)	14.2 % (19)	14.8 % (21)
H2	66.5 % (101)	68.7 % (92)	65.5 % (93)
H3	10.5 % (16)	10.5 % (14)	4.9 % (7)
H4	7.2 % (11)	6.7 % (9)	14.8 % (21)

The four haplotypes were determined in all three study groups. No significant differences were found in the distribution of the haplotypes. The significance level was set to P < 0.05

**Table 5:** Soluble endothelial protein C receptor (sEPCR) levels of hospitalized patients and village population

sEPCR Median [25/75]	Malaria patients 161.1 [124.4/213.4] N = 76		Non-malaria patients 130.6 [109.5/165.5] N = 67		Villages 238.0 [178.0/292.4] N = 71	
H1 vs. Hx	150.3 [135.0/200.6] N = 20 P = 0.6	165.6 [123.5/215.0] N = 56	115.6 [108.9/131.8] N = 19 P = 0.09	133.2 [109.8/208.8 N = 48	208 [173.0/247.0] N = 17 P = 0.09	259.0 [196.5/297.5] N = 54
H2 vs. Hx	160.3 [123.9/216.3] N = 66 P = 0.9	163.0 [128.9/195.2] N = 10	126.7 (108.9/154.6] N = 63 P = 0.06	215.3 [191.2/228.6] N = 4	247.0 [178.0/294.0] N = 61 P = 1.0	221.5 [1855.0/276.5] N = 10
H3 vs. Hx	193.2 [169.1/268.0] N = 16 *P* *=* *0.009*	153.3 [122.6/208.2] N = 60	236.7 [197.6/288.6] N = 13 *P* *<* *0.001*	119.4 [105.3/139.7] N = 54	493.0 [361.0/628.4] N = 6 *P* *=* *0.007*	232.0 [178.0/284.5] N = 65
H4 vs. Hx	156.6 [120.6/203.0] N = 10 P = 0.7	161.1 [125.4/213.6] N = 66	131.2 [120.6/169.4] N = 9 P = 0.7	128.7 [109.5/159.6] N = 58	231.3 [194.0/283.0] N = 19 P = 0.8	248.0 [177.8/298.6] N = 52

sEPCR levels were determined in all patients and village samples. Individuals who carry at least one H3 allele had higher levels of sEPCR than those carrying none. This was found in all three groups. Statistically significant differences were found in the general sEPCR level between all three groups. The non-malaria patient group differed from both the malaria and village groups by having lower levels of sEPCR (P = 0.001 and P < 0.001, respectively), and the malaria patient group differed from the village group by having lower levels of sEPCR (P < 0.001). The significance level was set to P < 0.05

The *PROCR* haplotypes and sEPCR levels of malaria patients were also analysed by comparing patients with severe or uncomplicated malaria (Table 6). No difference in frequency of the H3 haplotype was detected (Χ^2^ = 1.9, P = 0.2) or in sEPCR levels (P = 0.8). The cerebral malaria patients (N = 17) were then separated from the other severe cases (hyperparasitaemia, severe anaemia and respiratory distress) to determine if these patients differed in sEPCR level. No difference was found between the two groups (P = 0.7).

**Table 6:** Haplotype distribution and soluble endothelial protein C receptor (sEPCR) levels in severe and uncomplicated malaria patients

sEPCR Median [25/75]	Severe malaria patients 161.1 [125.2/213.4] N = 52		Uncomplicated malaria patients 157.4 [122.1/212.3] N = 24	
H1 vs. Hx	155.9 [128.7/215.6] N = 14 P = 0.9	161.1 [124.8/211.8] N = 38	146.7 [138.5/159.2] N = 6 P = 0.6	172.0 [121.0/226.8] N = 18
H1 (n)	16.4 % (17)		14.6 % (7)	
H2 vs. Hx	161.1 [125.5/216.3] N = 46 P = 0.3	145.2 [120.4/183.1] N = 6	146.0 [120.1/212.3] N = 20 P = 0.3	179.1 [155.6/264.8] N = 4
H2 (n)	66.4 % (69)		66.7 % (32)	
H3 vs. Hx	217.3 [169.1/318.8] N = 8 *P* *=* *0.03*	156.5 [123.5/208.4] N = 44	187.4 [165.3/242.5] N = 8 P = 0.1	135.6 [120.1/180.1] N = 16
H3 (n)	7.7 % (8)		16.7 % (8)	
H4 vs. Hx	134.1 [119.2/189.2] N = 9 P = 0.3	161.1 [125.6/215.4] N = 43	467.8 [467.8/467.8] N = 1 NA	153.8 [121.6/203.6] N = 23
H4 (n)	9.6 % (10)		2.1 % (1)	

The haplotype distribution and sEPCR levels are shown for the severe and uncomplicated malaria patients. A difference in sEPCR level was seen in individuals with at least one H3 allele compared to those with none, however, this was only seen in patients with severe and not uncomplicated malaria. No differences were found in the haplotype distributions or in the general sEPCR level between the two groups. The significance level was set to P < 0.05

**Fig. 2:**
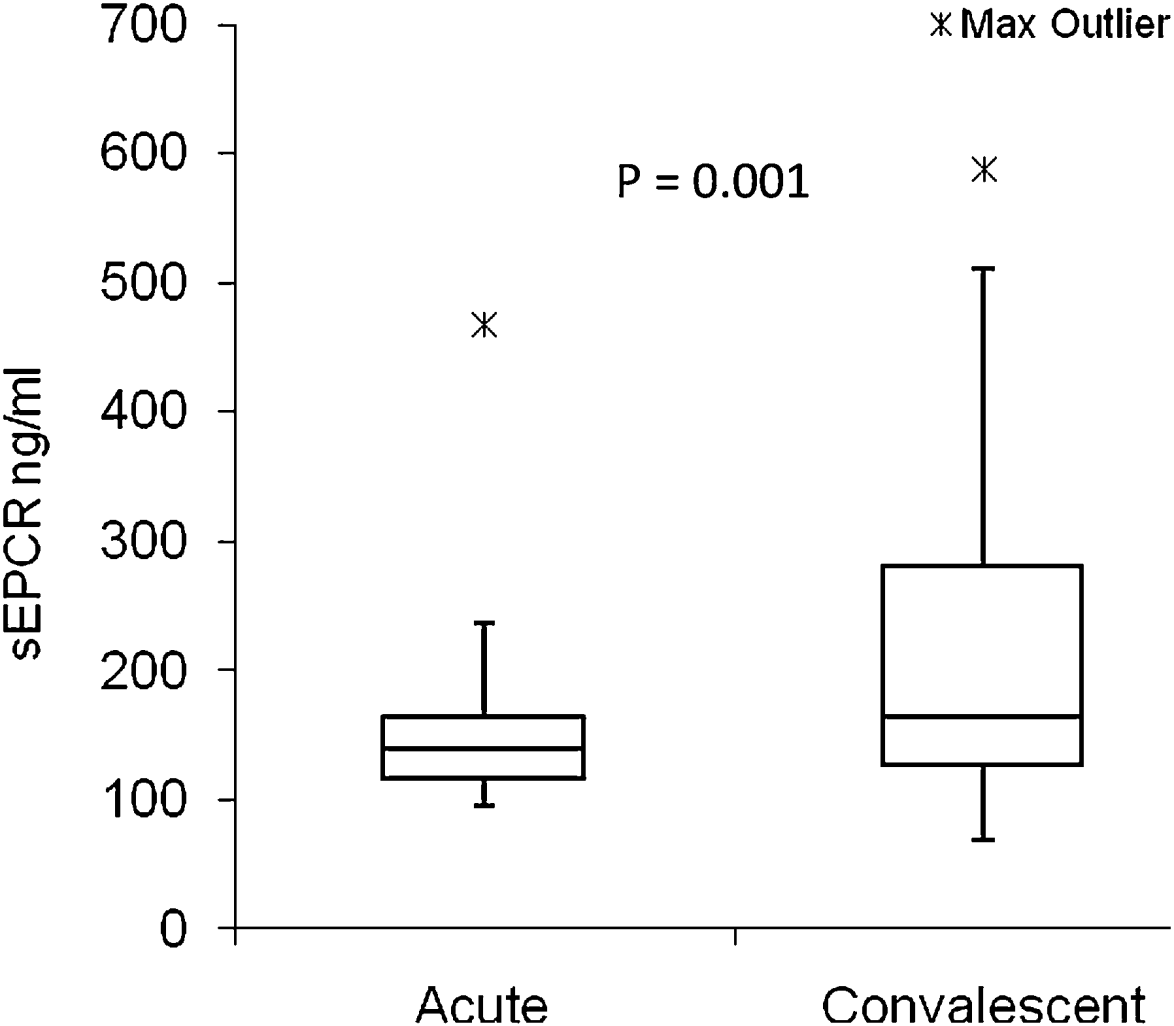
Soluble endothelial protein C receptor (sEPCR) levels in acute malaria patients and after follow-up. sEPCR levels were measured in acute ill malaria patients and after recovery (N = 28). The level of sEPCR was significantly lower (P = 0.001) during acute illness compared to the same patients after recovery. The significance level was set to P < 0.05

Finally, to investigate if there was any difference in sEPCR level during malaria and 3 weeks post-treatment, a sub-set of 28 children was included in a follow-up study (Fig. 2). The level of sEPCR was significantly higher during convalescence [median 163.8 ng/ml, (25/75 quartiles = 126.7/280.1)], than during acute infection [median 138.3 ng/ml, (25/75 quartiles = 116.6/164.3)], P = 0.001.

## Discussion

This study investigated the four defined EPCR haplotypes resulting from SNPs of the *PROCR* gene and their possible association with the level of sEPCR and disease outcome in Tanzanian patients infected with falciparum malaria. Based on earlier findings, it was hypothesized that the H3 haplotype, resulting in higher levels of sEPCR, could confer a protective advantage against severe malaria.

The haplotype distribution in this Tanzanian population differed from a Caucasian population [18], with a higher frequency of H2 (66.6 vs. 42.9 %) and lower frequency of H1 (15.0 vs. 39.4 %) while the frequency of H3 did not differ significantly between the two populations (8.6 vs. 12.7 %) [18]. SNPs of the *PROCR* gene have also been studied without defining these haplotypes in Ghana, Benin and Thailand [25–27]. In Ghana and Benin, very similar frequencies of the SNPs defining H1 (4868)C and H3 (6936G) were found compared to this Tanzanian study. In Ghana, the (4868)C was found at a frequency of 12.6 % compared to 16.3 % in this study. The (6936)G was found at frequencies of 5.9 % (Ghana) and 8.0 % (Benin) compared to the 9.9 % in Tanzania [25, 26]. Lastly, a Thai study [27] found a frequency of 30.5 % of the (6936)G, which is significantly higher than was found in Tanzania, Ghana and Benin [27].

In this study, the haplotypes were studied in three groups: malaria patients, patients with other infections and a village population. Malaria patients were divided into sub-groups based on severity of the disease and disease manifestation. No differences in H3 frequency were found between any of the groups. This indicates that the studied *PROCR* haplotypes are not a determining factor for severity of the disease, which is in concordance with findings from the study in Ghana, investigating over 40 SNPs, where none of the investigated SNPs was associated with severe malaria [26]. Both the present study and the Ghanaian study contradicts findings from the Thai study, where the (6936)G allele was associated with protection against severe malaria. However, this association was found when using a recessive inheritance model and not adjusting for multiple testing [27]. Applying a recessive inheritance model to the data of the study in Ghana found no association between (6936)G and protection either [26]. The study participants in the Thai study were significantly older than in this current study, and the contradictory findings may be biased if age has an effect on sEPCR level. Differences in selection pressure may also contribute to the differences found between populations.

The present study also examined the association between sEPCR plasma level and malaria symptoms. Non-malaria patients were found to have the lowest levels of sEPCR, whereas the village population had the highest, and no difference was seen between uncomplicated, severe or cerebral malaria patients. Increased sEPCR levels were found in individuals who had recovered from a malaria episode. These findings differ from the study in Benin, where high levels of sEPCR were associated with cerebral malaria and death [25], and surviving cerebral malaria patients had reduced sEPCR after 30 days to similar levels as seen for other severe and uncomplicated malaria patients [25]. One explanation for these differences could be that the patient group studied in Benin had progressed to a more severe state of malaria, perhaps causing acutely increased sEPCR levels, although this theory is compromised by the high sEPCR levels in the Tanzanian village population and that the Tanzanian patients recovering from malaria had an increase in sEPCR levels. Both these current findings and the findings from Benin are inconsistent with observations done in Malawi, where cellular-bound EPCR appeared to be reduced on endothelial cells with sequestered parasites, and sEPCR levels were lower in plasma but higher in cerebrospinal fluid in cerebral malaria patients compared to non-malaria febrile controls [28].

These discrepancies may be related to different disease state and ethnicity of patients, as well as the opposing effects that sEPCR binding to maturing circulating parasites [3], sEPCR shedding from endothelial cells, and immune acquisition may have on sEPCR plasma levels during disease.

As observed in previous studies [19, 22, 23, 25] this study showed that higher levels of sEPCR were associated with individuals with at least one H3 allele. Interestingly, a high plasma level of sEPCR was also seen in a sub-set of study participants with no H3 allele. In the Ghanaian study, the *PROCR* gene was sequenced and thoroughly examined to identify new SNPs [26]. However, sEPCR levels were not determined, and it is possible that some of these, or unidentified SNPs, are associated with elevated sEPCR plasma levels and protection against malaria; indeed, as suggested, any protective role of sEPCR may not be simply reflected by a haplotype-disease relationship.

PfEMP1 binds EPCR with high specificity and affinity, and with a 3D structure preserved across sequence diversity [4]. This interaction inhibits natural APC binding and is likely to aggravate pathogenesis of severe malaria. As PfEMP1 precludes APC binding by interaction at the same binding pocket site on EPCR as APC, it is unlikely that the EPCR could mutate to evade PfEMP1 binding without the detrimental loss of APC function. If protective mechanisms have developed in face of the PfEMP1–EPCR interaction, these could relate to the sEPCR level, e.g., through epigenetic control of *PROCR* expression. Thus, a good starting point would be larger studies of sEPCR levels in malaria-exposed populations to examine if elevated sEPCR levels do serve as a mechanism of protection against malaria.

## Conclusion

The four defined haplotypes of the *PROCR* gene were studied in relation to malaria and sEPCR level in a Tanzanian study population and hospitalized patients. Frequencies of SNPs determining *PROCR* haplotypes were in concordance with other African studies. *PROCR* H3 was associated with higher levels of sEPCR, confirming earlier findings, however, in this Tanzanian population; neither *PROCR* haplotype nor level of sEPCR was associated with severe malaria.

## Abbreviations

APC: activated protein C
EPCR: endothelial protein C receptor
H1–4: haplotype 1–4
LDR-FMA: ligase detection reaction—fluorescent microsphere assay
PAR-1: protease activated receptor 1
PCR: polymerase chain reaction
PfEMP1: p*lasmodium falciparum* erythrocyte membrane protein 1
sEPCR: soluble endothelial protein C receptor
SNP: single nucleotide polymorphism
WHO: World Health Organization
